# A scoping review for building a criticality-based conceptual framework of altered states of consciousness

**DOI:** 10.3389/fnsys.2023.1085902

**Published:** 2023-05-25

**Authors:** Charles Gervais, Louis-Philippe Boucher, Guillermo Martinez Villar, UnCheol Lee, Catherine Duclos

**Affiliations:** ^1^Department of Psychology, Université de Montréal, Montréal, QC, Canada; ^2^Centre for Advanced Research in Sleep Medicine & Integrated Trauma Centre, Centre Intégré Universitaire de Santé et de Services Sociaux du Nord-de-l’île-de-Montréal, Montréal, QC, Canada; ^3^Department of Neuroscience, Université de Montréal, Montréal, QC, Canada; ^4^Department of Biomedical Sciences, Université de Montréal, Montréal, QC, Canada; ^5^Department of Anesthesiology, University of Michigan Medical School, Ann Arbor, MI, United States; ^6^Center for Consciousness Science, University of Michigan Medical School, Ann Arbor, MI, United States; ^7^Department of Anesthesiology and Pain Medicine, Université de Montréal, Montréal, QC, Canada; ^8^CIFAR Azrieli Global Scholars Program, Toronto, ON, Canada

**Keywords:** criticality, consciousness, disorders of consciousness, altered states of consciousness, psychedelics, anesthesia, sleep, neuroimaging

## Abstract

The healthy conscious brain is thought to operate near a critical state, reflecting optimal information processing and high susceptibility to external stimuli. Conversely, deviations from the critical state are hypothesized to give rise to altered states of consciousness (ASC). Measures of criticality could therefore be an effective way of establishing the conscious state of an individual. Furthermore, characterizing the direction of a deviation from criticality may enable the development of treatment strategies for pathological ASC. The aim of this *scoping review* is to assess the current evidence supporting the criticality hypothesis, and the use of criticality as a conceptual framework for ASC. Using the PRISMA guidelines, Web of Science and PubMed were searched from inception to February 7th 2022 to find articles relating to measures of criticality across ASC. *N* = 427 independent papers were initially found on the subject. *N* = 378 were excluded because they were either: not related to criticality; not related to consciousness; not presenting results from a primary study; presenting model data. *N* = 49 independent papers were included in the present research, separated in 7 sub-categories of ASC: disorders of consciousness (DOC) (*n* = 5); sleep (*n* = 13); anesthesia (*n* = 18); epilepsy (*n* = 12); psychedelics and shamanic state of consciousness (*n* = 4); delirium (*n* = 1); meditative state (*n* = 2). Each category included articles suggesting a deviation of the critical state. While most studies were only able to identify a deviation from criticality without being certain of its direction, the preliminary consensus arising from the literature is that non-rapid eye movement (NREM) sleep reflects a subcritical state, epileptic seizures reflect a supercritical state, and psychedelics are closer to the critical state than normal consciousness. This scoping review suggests that, though the literature is limited and methodologically inhomogeneous, ASC are characterized by a deviation from criticality, though its direction is not clearly reported in a majority of studies. Criticality could become, with more extensive research, an effective and objective way to characterize ASC, and help identify therapeutic avenues to improve criticality in pathological brain states. Furthermore, we suggest how anesthesia and psychedelics could potentially be used as neuromodulation techniques to restore criticality in DOC.

## Introduction

Though scientists and philosophers have not established a single definition of consciousness, it is generally agreed that conscious beings are aware of themselves, their surroundings, and their own perception. As such, consciousness is thought to require the dual capacity for *wakefulness* (arousal) and *awareness* of oneself and the environment (Blume et al., 2015). In healthy individuals, wakefulness and awareness covary across sleep and wake states, whereas pathological states of consciousness, such as disorders of consciousness (DOC), may reflect a dissociation of the two.

The assessment of human consciousness generally relies on an individual’s willingness or capacity to reliably respond to the external environment at the time of assessment. However, consciousness can exist in unresponsive individuals (Owen et al., 2006; Sanders et al., 2012), and behavioral assessments have been shown to yield low diagnostic sensitivity in unresponsive individuals (Schnakers et al., 2009) suggesting they are insufficient to i) detect capacity for consciousness, and ii) probe the neural processes capable of sustaining consciousness. Major theories of consciousness disagree on the key neural substrates of conscious awareness (Lamme, 2010; Tononi et al., 2016; Mashour et al., 2020), and to this day, no single brain region has been unequivocally identified as the seed of consciousness. Alternatively, consciousness “might emerge through the complex interactions of spatially and temporally distributed brain functions” (Lee and Mashour, 2018). In this perspective, the brain can be conceived as a complex network of structurally and/or functionally interacting components. Recent projects in neuroimaging and machine learning have produced highly accurate methods of assessing and predicting consciousness in unresponsive individuals (Logi et al., 2011; Casali et al., 2013; Stender et al., 2014, 2015; Wu et al., 2015; Chennu et al., 2017; Song et al., 2018; Duclos et al., 2022). However, the majority of these indices rely on specialized technologies (e.g., fMRI, TMS) that have contraindications for many pathologically-unconsciousness individuals, preventing their widespread adoption for the assessment of consciousness. Consequently, there is a critical need to understand which functional properties of the brain give rise to human consciousness, and to develop accessible technologies for detecting consciousness using bedside techniques.

The criticality hypothesis of consciousness proposes that a healthy conscious brain self-organizes into a critical state, or critical point (Wilting and Priesemann, 2019), which optimizes information processing (Shew and Plenz, 2013; Scott et al., 2014; Fagerholm et al., 2016; Kim and Lee, 2019). Conversely, deviations from this critical state are hypothesized to give rise to both pathological (e.g., coma) and non-pathological (e.g., sleep) altered states of consciousness (ASC), as well as a reduction in the brain’s optimal information processing capacity. The criticality hypothesis therefore offers a potentially unifying theory of the functional mechanisms underlying consciousness and provides quantifiable empirical measures that can be applied to the brain. However, the application of criticality to neuroscience remains recent, and it is unclear whether the criticality hypothesis is currently supported by the scientific literature.

The concept of criticality was originally introduced in statistical mechanics to study phase transitions. A phase transition is a change in the state of a system, such as from liquid to gas, that occurs when a certain critical value of a parameter, such as a temperature, is reached. Criticality is a state of a system that is on the verge of a phase transition (Cocchi et al., 2017; Muñoz, 2018). At a critical point, small fluctuations in the system are not damped, but instead, propagate throughout the system at all spatial and temporal scales. This results in scale-free (power-law) fluctuations in both the spatial and temporal domains, as well as high susceptibility to external stimuli. For example, when water is at its freezing point, a small change in temperature can cause the entire system to freeze or melt. This is because the system is at a critical point, and small fluctuations can propagate throughout the system and cause a large-scale change in state. The state of a system in which the control parameter is below a critical point is called a subcritical state. In contrast, the state of a system in which the control parameter is above a critical point is called a supercritical state. Different phase transitions can be investigated depending on the control parameter of interest, such as at the edges of chaos, synchrony, or avalanche. Criticality measures, such as spatial or temporal correlation, complexity, synchronization, or neuronal avalanche, can be used to determine the system state (subcritical, critical, or supercritical). In the context of biological systems, criticality in the brain confers many functional advantages that may establish a functional foundation for the emergence of consciousness. These advantages include large computational capability with temporal correlation (memory) and long-range correlation (efficient brain integration), high flexibility to adapt to a changing environment from scale-invariance, and wide repertoires of brain states from large fluctuations. Therefore, recent studies have proposed brain criticality as a necessary condition for the emergence of consciousness, and deviations from the critical state (i.e., after a transition to one of the possible states in a sub- or supercritical state) are hypothesized to give rise to ASC (Kim and Lee, 2019; Lee et al., 2019; Kim M. et al., 2021).

### An overview of measures of criticality in the brain

Quantitative evaluation of criticality in the brain is crucial to determine whether a brain is operating near or far from a critical state. The measures are developed primarily to quantify the typical system characteristics near criticality: scale-invariance (e.g., power-law of avalanche sizes/intervals, hurst exponent, branching parameter), large spatiotemporal correlations (e.g., long-range temporal correlations, autocorrelation), and large fluctuations (e.g., detrended fluctuation analysis).

One of the most common methods used is the analysis of neuronal avalanches. The basis of this method lies in the assumption that neuronal networks work in a similar fashion to nuclear chain reactions and earthquakes, where a simple unit reaches a threshold and then propagates its activity to the rest of the system, thereby initiating an “avalanche”. These avalanches are known to follow a power-law distribution which can be used to determine criticality by evaluating the balance between rapidly dying activity and amplified activity over time (Beggs and Plenz, 2003; Beggs and Timme, 2012). Subsequently, it is possible to extract the power-law exponent of the neuronal avalanche by plotting the power-law dataset into a log-log graphic that will then produce a straight line with the slope being the exponent.

Another group of methods consist of the analysis of long-range temporal correlations, which focuses on the temporal structure of neuronal oscillations (Linkenkaer-Hansen et al., 2001). Different sub-methods exist for measuring these long-range temporal correlations, such as the autocorrelation function, which measures the degree at which a signal is similar to itself over time. Long-range temporal correlations are confirmed if the autocorrelation function decreases, as a function of time, according to a power-law with an exponent between -1 and 0, showing that even though the signal is changing over time, a certain portion stays coherent to the past signal (Meisel et al., 2017a). However, autocorrelation function is said to be greatly affected by trends and shows a disturbingly high amount of noise, especially for large time lags (Meisel et al., 2017a).

The next method, detrended fluctuation analysis, was introduced to resolve the issues of the autocorrelation function. Detrended fluctuation analysis produces a scaling exponent from the amplitude envelope of an oscillatory signal that, when between 0.5 and 1, indicates the presence of long-range temporal correlations in the time signal studied (Hardstone et al., 2012). Another popular method for measuring long-range temporal correlations is the Hurst exponent, which reflects the change in autocorrelation in a time series. A Hurst exponent between 0.5 and 1 confirms that long-range temporal correlations are present (Witton et al., 2019).

The last method described here for measuring long-range temporal correlations, is the pair correlation function, a measure of the variance of order parameters (i.e., phase coherence) between oscillators in a network (Yoon et al., 2015). Pair correlation function is maximal at a critical point, presenting a long-range correlation and high network susceptibility to external and internal stimulation (Yoon et al., 2015). However, the pair correlation function diminishes as a system deviates from criticality (Kim and Lee, 2019).

Another method used in the criticality literature is the branching parameter *m*, a stochastic description of the propagation of action potentials in a neuronal network. The branching parameter *m* stands for the mean number of subsequent spikes triggered after applying an extra spike to an excitatory neuron in the neuronal network. An *m* of 1 is found in the critical state while a greater *m* suggests a supercritical state and a lower *m* a subcritical state (Priesemann et al., 2019).

### Aim

The criticality hypothesis could be tested to elucidate the functional brain dynamics that underly various states of pathological and non-pathological consciousness. Considering that the brain of a healthy, awake subject is likely to operate in a lightly subcritical state (Priesemann et al., 2014), measuring the deviations from criticality could also be a promising way to refine the diagnosis and prognosis of pathological ASC such as DOC (Priesemann et al., 2013; Atasoy et al., 2017; Fekete et al., 2018; Hagemann et al., 2021; O’Byrne and Jerbi, 2022). A nuanced understanding of criticality and its association to consciousness may also provide insight into the potential therapeutic avenues that could help restore criticality, and possibly consciousness, in pathological ASC.

Our aim in this review is to determine the scope of the literature on criticality in ASC, and to identify the evidence currently supporting the criticality hypothesis in these states of consciousness, in both humans and animals. Although some reviews already exist concerning brain criticality and its applications to consciousness (Zimmern, 2020; Walter and Hinterberger, 2022), this review aims to evaluate how brain criticality changes throughout alterations in consciousness across humans and animals, and to provide a conceptual framework for the implementation of criticality as a tool for evaluation and treatment of ASC.

## Materials and methods

### Design

Considering the broad and heterogeneous study subject, and the presence of multiple subtopics in the field of consciousness, which was unamenable to a systematic review, we conducted a scoping review of the literature. This study followed the Preferred Reporting Items for Systematic Review and Meta-Analyses extension for scoping reviews (PRISMA-ScR) (Tricco et al., 2018), and focused its search on published studies available through open access or academic research databases.

### Search strategy

Two databases (Web of Science, PubMed) were searched from inception to the 7th of February 2022 to find articles relating to measures of criticality across different ASC. The following keywords were used during the search of the two databases: “criticality sleep”, “criticality coma”, “criticality unresponsive wakefulness syndrome”, “criticality sedation”, “criticality anesthesia”, “criticality disorders of consciousness”, “criticality epileptic seizure”, “criticality meditation”, “criticality delirium”, “criticality rem”, “criticality nrem”, “criticality minimally conscious state”, “criticality consciousness”, “criticality vegetative state”, “criticality psychedelic”, “criticality eeg” and “criticality fmri”. To ensure the maximum number of articles on the subject were found, the searches were automatically upgraded by the respective algorithm of each database (see Table 1 for the complete details of the search in PubMed). In all databases, the search was limited to studies published in English or French.

**TABLE 1:** Complete electronic research of the PubMed database.

Database	PubMed
**Strategy**	
Criticality sleep	(”criticalities”[All Fields] OR “criticality”[All Fields]) AND (”coma”[MeSH Terms] OR “coma”[All Fields])
Criticality coma	(”criticalities”[All Fields] OR “criticality”[All Fields]) AND (”sleep”[MeSH Terms] OR “sleep”[All Fields] OR “sleeping”[All Fields] OR “sleeps”[All Fields] OR “sleep s”[All Fields])
Criticality unresponsive wakefulness syndrome	(”criticalities”[All Fields] OR “criticality”[All Fields]) AND (”unresponsive”[All Fields] OR “unresponsiveness”[All Fields]) AND (”wakeful”[All Fields] OR “wakefulness”[MeSH Terms] OR “wakefulness”[All Fields] OR “wakes”[All Fields] OR “waking”[All Fields] OR “wakings”[All Fields]) AND (”syndrom”[All Fields] OR “syndromal”[All Fields] OR “syndromally”[All Fields] OR “syndrome”[MeSH Terms] OR “syndrome”[All Fields] OR “syndromes”[All Fields] OR “syndrome s”[All Fields] OR “syndromic”[All Fields] OR “syndroms”[All Fields])
Criticality sedation	(”criticalities”[All Fields] OR “criticality”[All Fields]) AND (”sedate”[All Fields] OR “sedated”[All Fields] OR “sedating”[All Fields] OR “sedation”[All Fields] OR “sedations”[All Fields])
Criticality anesthesia	(”criticalities”[All Fields] OR “criticality”[All Fields]) AND (”anaesthesia”[All Fields] OR “anesthesia”[MeSH Terms] OR “anesthesia”[All Fields] OR “anaesthesias”[All Fields] OR “anesthesias”[All Fields])
Criticality disorders of consciousness	(”criticalities”[All Fields] OR “criticality”[All Fields]) AND (”disease”[MeSH Terms] OR “disease”[All Fields] OR “disorder”[All Fields] OR “disorders”[All Fields] OR “disorder s”[All Fields] OR “disordes”[All Fields]) AND (”consciously”[All Fields] OR “consciousness”[MeSH Terms] OR “consciousness”[All Fields] OR “consciousnesses”[All Fields])
Criticality epileptic seizure	(”criticalities”[All Fields] OR “criticality”[All Fields]) AND (”seizures”[MeSH Terms] OR “seizures”[All Fields] OR (”epileptic”[All Fields] AND “seizure”[All Fields]) OR “epileptic seizure”[All Fields])
Criticality meditation	(”criticalities”[All Fields] OR “criticality”[All Fields]) AND (”meditate”[All Fields] OR “meditated”[All Fields] OR “meditating”[All Fields] OR “meditation”[MeSH Terms] OR “meditation”[All Fields] OR “meditations”[All Fields] OR “meditational”[All Fields] OR “meditative”[All Fields] OR “meditator”[All Fields] OR “meditators”[All Fields])
Criticality fmri	(”criticalities”[All Fields] OR “criticality”[All Fields]) AND (”magnetic resonance imaging”[MeSH Terms] OR (”magnetic”[All Fields] AND “resonance”[All Fields] AND “imaging”[All Fields]) OR “magnetic resonance imaging”[All Fields] OR “fmri”[All Fields])
Criticality delirium	(”criticalities”[All Fields] OR “criticality”[All Fields]) AND (”delirium”[MeSH Terms] OR “delirium”[All Fields] OR “deliriums”[All Fields])
Criticality rem	(”criticalities”[All Fields] OR “criticality”[All Fields]) AND (”rangelecolmanag”[Journal] OR “rem”[All Fields])
Criticality nrem	(”criticalities”[All Fields] OR “criticality”[All Fields]) AND “nrem”[All Fields]
Criticality minimally conscious state	(”criticalities”[All Fields] OR “criticality”[All Fields]) AND (”persistent vegetative state”[MeSH Terms] OR (”persistent”[All Fields] AND “vegetative”[All Fields] AND “state”[All Fields]) OR “persistent vegetative state”[All Fields] OR (”minimally”[All Fields] AND “conscious”[All Fields] AND “state”[All Fields]) OR “minimally conscious state”[All Fields])
Criticality consciousness	(”criticalities”[All Fields] OR “criticality”[All Fields]) AND (”consciously”[All Fields] OR “consciousness”[MeSH Terms] OR “consciousness”[All Fields] OR “consciousnesses”[All Fields])
Criticality vegetative state	(”criticalities”[All Fields] OR “criticality”[All Fields]) AND (”persistent vegetative state”[MeSH Terms] OR (”persistent”[All Fields] AND “vegetative”[All Fields] AND “state”[All Fields]) OR “persistent vegetative state”[All Fields] OR (”vegetative”[All Fields] AND “state”[All Fields]) OR “vegetative state”[All Fields])
Criticality psychedelic	(”criticalities”[All Fields] OR “criticality”[All Fields]) AND (”hallucinogens”[Pharmacological Action] OR “hallucinogens”[MeSH Terms] OR “hallucinogens”[All Fields] OR “psychedelic”[All Fields] OR “psychedelics”[All Fields])
Criticality eeg	(”criticalities”[All Fields] OR “criticality”[All Fields]) AND (”electroencephalography”[MeSH Terms] OR “electroencephalography”[All Fields] OR “eeg”[All Fields])

### Inclusion and exclusion criteria

All primary studies including case reports, case studies, clinical trials, cross-sectional studies and randomized controlled trials exploring criticality in an altered state of consciousness (i.e., DOC, sleep, anesthesia, epilepsy, psychedelics and shamanic state of consciousness, meditation, delirium) were considered for this review. Reviews, commentaries, opinion papers and book chapters were not considered for inclusion.

Considering that the degree of evidence of criticality in ASC is still arguably low, a broad population of interest was agreed on for this study. Studies looking at brain criticality in humans and animals were included. Studies on criticality in healthy conscious wakefulness or during cognitive tasks were excluded in both humans in animals. Studies looking at modelized brain data were also excluded. No other exclusion criteria were applied.

To be included in this review article, studies had to look at brain criticality in ASC. No limitations were placed on the methods used to measure brain criticality as long as the method was recognized and well documented to measure criticality.

### Study selection

**FIGURE 1:**
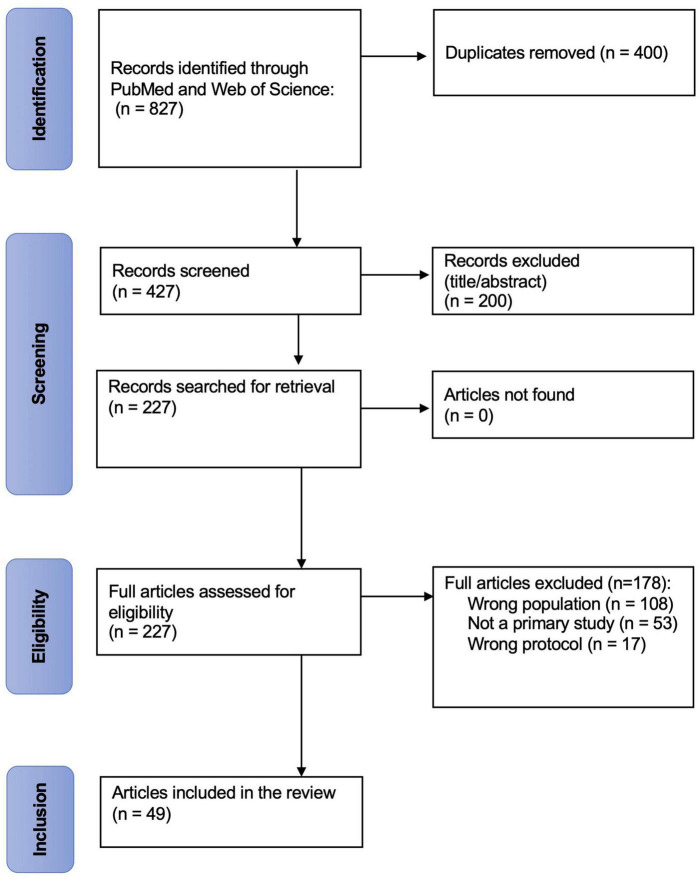
PRISMA-ScR flow chart. This flow chart depicts the flow stages of the screening process and articles retrieved, screened and included at various stages of the review process.

Following the search, all identified studies were uploaded into Covidence^®^ for duplicate elimination and further screening. Subsequently, article titles and abstracts generated from the initial search were independently assessed for eligibility by 3 members of the review team (CD, LPB, GMV) based on the inclusion and exclusion criteria mentioned previously. Two votes were necessary for an article to go through the next stage of selection. The full texts of remaining articles were independently examined by CD and LPB to reach a final list of articles. Disagreements at either screening stage were resolved through discussion with the supervisor of the study (CD). Consensus was reached for all included articles. The reasons for study exclusion were documented (Figure 1).

### Data extraction

The review team identified the main areas of interest as follows: (1) title; (2) authors; (3) publication date; (4) DOI; (5) type of study; (6) goal of the study; (7) criticality measures used; (8) methodology; (9) main results. For each study, relevant data were extracted using a customized data extraction form in a Microsoft Excel spreadsheet. All data were extracted by LPB and verified by CD and CG for accuracy and quality.

## Results

A total of *N* = 427 independent papers were initially found on the topic of interest. *N* = 378 were excluded because they were either: not related to criticality; not related to consciousness; not presenting results from a primary study; presenting modelized data rather than actual data from human or animal recordings. Finally, *n* = 49 independent papers were included in the present review, separated in 7 sub-categories of ASC: sleep (*n* = 13); anesthesia (*n* = 18), DOC and coma, including vegetative state (or unresponsive wakefulness syndrome) and minimally conscious state (*n* = 5); epilepsy (*n* = 12); psychedelics and shamanic state of consciousness (*n* = 4); delirium (*n* = 1); meditation (*n* = 2). Some articles (Ribeiro et al., 2010; Liu et al., 2014; Lee et al., 2019; Fekete et al., 2021; Kim M. et al., 2021) were included in more than 1 sub-category as they presented results concerning various ASC. *N* = 33 studies were conducted in human participants, *n* = 15 were conducted in animal subjects and *n* = 1 study had both animal and human subjects. Reported results are from a human population unless mentioned otherwise.

### Characteristics of included studies

**FIGURE 2:**
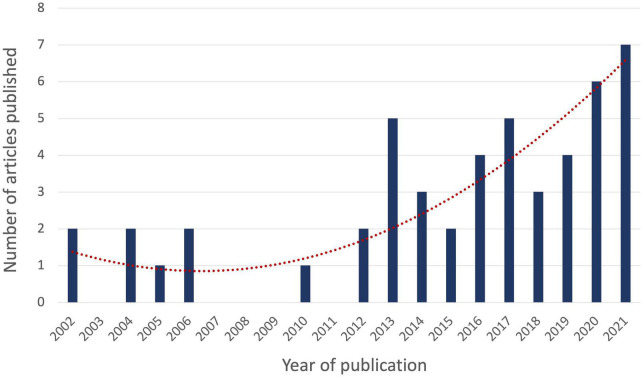
Year of publication of articles relating to criticality in ASC. The number of articles on criticality in an altered state of consciousness (ASC) published per year is depicted by the vertical blue bars. The exponential trend line is depicted in dotted red.

The literature search yielded articles published from October 2002 to February 7^th^ 2022, with the majority (*n* = 27, 55%) being published in the last 5 years. The years 2020 and 2021 have seen the most publications (*n* = 6 for 2020 and *n* = 7 for 2021, together representing 26.5% of all articles published) after a constant increase from the years prior, showing a growing interest in the application of criticality to ASC (Figure 2).

The articles included in this review used various methods of measurement of criticality. The two most common were the method of neuronal avalanche (*n* = 21, 43%) and long-range temporal correlations (*n* = 13, 27%). Other methods used are the pair correlation function (*n* = 3, 6%), a stability parameter (*n* = 2, 4%), a branching parameter *m* (*n* = 1, 2%) and phase lag entropy (*n* = 1, 2%). *N* = 8 articles (16%) reported methods that were less common and more personalized to the research team that used them (Table 2).

**TABLE 2:** Methods used to measure criticality in articles included in this review.

Methods	Definition	*N* articles	Articles
**Neuronal avalanche**	Propagation of neuronal activity following a power-law (Beggs and Plenz, 2003).	21	Cranstoun et al., 2002; Worrell et al., 2002; Li et al., 2005; Freeman et al., 2006; Ribeiro et al., 2010; Dehghani et al., 2012; Meisel et al., 2012, 2013; Priesemann et al., 2013; Scott et al., 2014; Arviv et al., 2016; Fagerholm et al., 2016, 2018; Hudetz et al., 2016; Hahn et al., 2017; Fekete et al., 2018, 2021; Bocaccio et al., 2019; Dürschmid et al., 2020; Meisel, 2020; Varley et al., 2020b
**Long-Range Temporal Correlations (LRTC)**	Global term for temporal structure of neuronal oscillations (Linkenkaer-Hansen et al., 2001).	13 (combined)	
Autocorrelation function	Correlation of a single signal over time (Meisel et al., 2017a).	2	Meisel et al., 2017b; Kim H. et al., 2021
Detrended fluctuation analysis	Scaling exponent from the amplitude envelope of an oscillatory signal (Hardstone et al., 2012).	9	Parish et al., 2004; Monto et al., 2007; Allegrini et al., 2013, 2015; Paradisi et al., 2013; Krzemiński et al., 2017; Irrmischer et al., 2018; Wang et al., 2019; Lombardi et al., 2020
Hurst exponent	Change in autocorrelation over time (Witton et al., 2019).	2	Tagliazucchi et al., 2013; Yan et al., 2016
**Pair Correlation Function**	Variability of the network (Kim et al., 2017).	3	Kim and Lee, 2019; Huels et al., 2021; Kim M. et al., 2021
**Branching parameter m**	Capacity to propagate activity in a network (Priesemann et al., 2019).	1	Hagemann et al., 2021
**Stability parameter**	Measure of the dynamical instability seen at or near the critical state (Solovey et al., 2012).	2	Alonso et al., 2014; Solovey et al., 2015
**Phase Lag Entropy (PLE)**	Quantifies the diversity of temporal patterns of the phase relationship between two signals (Lee et al., 2019).	1	Lee et al., 2019
**Other methods**		8	Liu et al., 2014; Scale-free distributions of node size and degrees Stramaglia et al., 2017; Structure-function relation Cerf et al., 2004; Parameter of fluctuation of electropathophysiological activity Abeyasinghe et al., 2020; Dimensionality Atasoy et al., 2017; Power-laws of brain harmonics Ponce-Alvarez et al., 2022; Scaling parameter in fMRI Pullon et al., 2022; Long-time scale mutual information Varley et al., 2020a; Fractal dimension of cortical activity in temporal and spatial domains

In the following sections, we will briefly introduce each ASC and present articles found within this scoping review.

### Sleep

All animal species experience daily but reversible loss in their consciousness through their sleep. During sleep, wakefulness is entirely suspended and awareness is partially, but not completely dissolved. For example, both internal (e.g., pain) and external (e.g., alarm clock) signals can interrupt sleep, reflecting some level of awareness. Sleep is composed of rapid-eye movement sleep (REM), and non-rapid-eye movement sleep (NREM), which can further be subdivided into 3 stages (N1, N2, N3) reflecting sleep depth, and the degree to which awareness is temporarily lost. Each sleep stage has distinct electrophysiological characteristics that can be detected by EEG. Sleep depth is generally quantified by the density of slow oscillations within the brain, reflecting synchronized activity between large populations of neurons (Niethard et al., 2018). Although normal sleep is not considered to be a conscious state, some sleepers may experience some forms of consciousness during sleep. This is true in lucid dreaming, where the sleeper becomes aware that their perception is a dream, and may have some degree of control over dream elements. It is also the case for somnambulism, where the sleeper appears awake, but sensory perceptions are drastically decreased, such that sight, sounds, smell and pain are not fully perceived and experiences are not carried into the waking state (Popat and Winslade, 2015). Sleep is essential to overall health, learning, plasticity and neurogenesis (Walker and Stickgold, 2004; Tononi and Cirelli, 2014), and is likely to play a direct role in recovery from illness and injury.

In human and animal studies, criticality does not seem to be lost in sleep in general, except for NREM sleep, where data suggests a deviation from the critical state (Table 3). In three different studies that used detrended fluctuation analysis to measure long-range temporal correlations, it was found that deep sleep comes with a deviation from criticality (Allegrini et al., 2013, 2015; Paradisi et al., 2013), and, using Hurst exponent in fMRI, another study suggests that deep sleep is significantly associated with an exponentially decaying autocorrelation, meaning an even more subcritical brain than what is seen in wakefulness (Tagliazucchi et al., 2013). In rats, the same deviation from criticality in NREM sleep was found multiple times, whether using detrended fluctuation analysis to measure long-range temporal correlations (Wang et al., 2019; Lombardi et al., 2020) or by looking at neuronal avalanches (Freeman et al., 2006; Ribeiro et al., 2010). These results were also reproduced in humans using the autocorrelation function to measure long-range temporal correlations (Meisel et al., 2017b). Another evidence supporting that NREM sleep is associated with a deviation from criticality comes from a fMRI study that looked at the differences in the power-law of neuronal avalanches in every sleep stage and found that N2 induced the greatest deviation from power-laws found in wakefulness (Bocaccio et al., 2019). Although some studies seem to be converging on this topic, contradicting results were seen in a single study where NREM sleep was found to restore criticality by bringing dynamics closer to the critical point (Priesemann et al., 2013). Also, while studying the effect of sleep deprivation on neuronal avalanches and criticality, prolonged wakefulness was found to create a deviation from criticality (Freeman et al., 2006; Meisel et al., 2013). Sleep after these long bouts of wakefulness was discovered to restore criticality in the brain, pointing in the direction that sleep is involved in the self-organization of criticality (Freeman et al., 2006; Meisel et al., 2013). On the other hand, using local field potentials, an invasive recording method consisting of the insertion of micro-electrodes directly into the cerebral cortex, a study was not able to confirm any signs of power-law scaling while analyzing the neuronal avalanches recorded in the motor cortex of cats and monkeys, as well as the premotor and temporal cortex of epileptic humans during slow wave sleep as well as REM sleep (Dehghani et al., 2012).

**TABLE 3:** Results of studies on criticality in sleep.

References	Population	Cerebral activity measure	Criticality measure	Main findings
Freeman et al., 2006	1 epileptic human with intracranial electrodes on the right inferior temporal gyrus	iEEG	Neuronal avalanche	• Slow-wave sleep is accompanied by a loss of scale-free activity, thus deviating from criticality. • Increased synaptic input in the awake state seems to shift neocortex away from criticality Diminished input in slow wave sleep allows return toward criticality, but with some added risk of instability and seizures.
Ribeiro et al., 2010	14 rats with electrodes in V1, S1 and hippocampus	MEA/LFP	Neuronal avalanche	• SWS is associated with a deviation from a power-law distribution.
Dehghani et al., 2012	2 cats with electrodes in M1; 3 monkeys with electrodes in M1 and PMd; 2 humans with pharmaco-resistent epilepsy with electrodes in middle temporal gyrus	LFP	Neuronal avalanche	• There is an absence of power-law scaling of neuronal avalanches in all examined recordings, including SWS and REM sleep.
Allegrini et al., 2013	29 whole-night human recordings	hdEEG	Neuronal avalanche	• In NREM sleep, criticality breaks down and is restored during REM sleep.
Meisel et al., 2013	8 healthy adults during 40h of sustained wakefulness followed by sleep	EEG	Neuronal avalanche	• Sleep restriction creates a progressive distance from criticality toward states characterized by an imbalance toward excitation (supercritical). • Sleep shows to be restoring the critical state by recovering power-law characteristics.
Paradisi et al., 2013	29 whole-night recordings	EEG	DFA	• SWS is accompanied by a deviation of criticality using DFA.
Priesemann et al., 2013	5 adults with refractory partial epilepsy presurgical evaluation	LFP	Neuronal avalanche	• Neuronal avalanches differ depending on the vigilance states: SWS shows large avalanches, wakefulness shows intermediate avalanches, and REM sleep shows small avalanches. • Authors suggest that SWS is closest to the critical state.
Tagliazucchi et al., 2013	51 non-sleep deprived subjects	EEG/fMRI	Hurst exponent	• N2 and N3 show a decreased temporal complexity in specific brain regions using BOLD spontaneous fluctuations display.
Allegrini et al., 2015	29 whole-night human recordings	hdEEG	DFA	• While neuronal avalanches seem to be qualitatively unchanged in N2 and N3, a deviation from criticality is found using DFA. • The authors suggest that a new kind of self-organized criticality emerges in sleep, characterized by the absence of thermodynamical feedback of the order on the control parameter, making way for a more auto-organized system.
Meisel et al., 2017b	23 rats	LFP	ACF	• The long timescales found in wake and REM sleep are abolished during NREM sleep, which may explain the lack of responsiveness and loss of consciousness in this state.
Bocaccio et al., 2019	58 non-sleep deprived subjects	EEG/fMRI	Neuronal avalanche	• There is a significant effect of sleep stage on the scaling parameters of the cluster size power-law distributions. *Post hoc* statistical tests show that differences are maximal between wakefulness and N2 sleep.
Wang et al., 2019	20 rats: 10 controls and 10 with lesions of the parafacial zone (PZ)	PSG	DFA	• Bursts in θ and δ rhythms exhibit a complex temporal organization, with long-range power-law correlations and a robust duality of power law and exponential-like duration distributions, typical features of systems self-organizing at criticality.
Lombardi et al., 2020	10 rats	EEG	DFA	• The presence of transient bursts in θ and δ cortical rhythms is found during the sleep-wake cycle. These bursts exhibit a complex temporal organization typical of non-equilibrium systems self-organizing at criticality. • An anti-correlation between θ and δ bursts is found throughout the sleep-wake cycle, a further sign of critical behavior.

ACF, autocorrelation function; DFA, detrended fluctuation analysis; EEG, electroencephalography; fMRI, functional magnetic resonance imaging; hdEEG, high-density electroencephalography; iEEG, intracranial electroencephalography; LFP, local field potential; MEA, multielectrode arrays; M1, primary motor area; OSA, obstructive sleep apnea; PMd, dorsal premotor cortex; S1: primary somatosensory cortex; V1, primary visual area.

### Anesthesia

Anesthesia is a pharmacologically-induced and reversible state of unconsciousness. For surgical procedures, balanced anesthesia aims to create a synergistic state of analgesia, hypnosis, amnesia and muscle paralysis. Contrary to sleep, anesthesia is thought to be marked by a complete loss of both wakefulness and awareness, with a complete absence of responsiveness to commands and to pain. On the EEG, anesthesia is typically characterized by slow oscillations, similar to slow-wave (N3) sleep (Chander et al., 2014; Purdon et al., 2015). When used in lighter doses, anesthetic agents can induce sedation, ranging from minimal (e.g., drowsiness and relaxation) to deep (e.g., purposeful response to painful stimulation only). Anesthesia is often used as a model of unconsciousness and has been shown to reduce higher-order information processing, while not affecting primary sensory cortices (Hudetz and Mashour, 2016). Though previous studies suggest that both sleep and anesthesia may share common mechanisms, such as alterations in cortico-cortical and thalamocortical connectivity (Larson-Prior et al., 2009; Stamatakis et al., 2010; Heine et al., 2012), a lack of consensus persists regarding the neural mechanisms by which anesthetic agents induce unconsciousness.

A wide range of anesthetics have been used to study brain criticality in humans under anesthesia, and all results point in the direction that anesthesia induces a deviation from the critical state (Table 4). Studies using isoflurane and ketamine show a reduction in pair correlation function (Kim M. et al., 2021), as well as a reduction in phase lag entropy (Lee et al., 2019), meaning a deviation from criticality. Studies looking at general anesthesia under propofol show similar results. It was found that propofol induces greater stability properties in the neuronal system, which can be interpreted as a deviation from criticality considering that a recent model exhibiting complex spatio-temporal dynamics proposed that criticality emerges from dynamical instability (Alonso et al., 2014). Another study showed that the structure-function relation of the large scale intrinsic connectivity network is stronger under propofol anesthesia than what is seen in an awake resting state without anesthesia, which also indicates a deviation from criticality, as it has been shown that the functional pattern is less dependent on the underlying structural network at the critical state (Stramaglia et al., 2017). Another study that characterized criticality during propofol anesthesia in humans showed a decrease in complexity (as measured by Jensen-Shannon divergence) and a reduction of long-range temporal correlations at loss of consciousness, still pointing in the direction of a deviation from criticality (Pullon et al., 2022). Lastly, while trying to create a parallel between criticality and the Integrated Information Theory (Tononi, 2004), one group showed that, as a subject loses consciousness after injection of sevoflurane, criticality measured by pair correlation function and Φ, a surrogate metric of integrated information, both decrease, yielding further evidence that anesthesia induces a deviation from the critical state in humans (Kim and Lee, 2019). Lastly, a single study was found on criticality during propofol sedation. This study showed that the scale-free distributions of node size and degree (size being the number of contiguous voxels sharing a hemodynamic profile and degree being the number of node connections) were maintained before, during and after sedation, suggesting that criticality is maintained during sedation (Liu et al., 2014).

**TABLE 4:** Results of studies on criticality in anesthesia.

Refrences	Population	Cerebral activity measure	Criticality measure	Main findings
Ribeiro et al., 2010	14 rats with electrodes in HP, S1 and V1	MEA/LFP	Neuronal avalanche DFA	• The size distribution of neuronal avalanches follows a power-law distribution in awake animals and deviate from power-laws in the anesthesia group.
Alonso et al., 2014	3 human subjects receiving propofol anesthesia	ECoG	Stability properties of neuronal dynamics	• As the subject becomes anesthetized, there is an increase in the stability of neuronal dynamics, most prominently observed for high frequency oscillations. This stabilization suggests a deviation from criticality.
Liu et al., 2014	7 healthy human subjects receiving propofol as a sedative	fMRI	Scale-free distributions of functional node size and degrees	• Scale-free distributions of node size and node degrees are present across wakefulness, propofol sedation, and recovery. despite significant propofol-induced functional connectivity changes. • Significant changes in functional connectivity are found during propofol sedation.
Scott et al., 2014	3 mice receiving pentobarbital as an anesthetic	Optic voltage imaging	Neuronal avalanche	• As mice recover from anesthesia, scale-free spatiotemporal patterns of neuronal activity characteristic of criticality gradually emerge. • In contrast, cortical dynamics of anesthetized mice are not scale-free, suggesting a deviation from criticality in anesthesia.
Solovey et al., 2015	4 male monkeys receiving, in separate time-frames, propofol and ketamine-medetomidine	ECoG	Stability parameter	• Reversible loss of consciousness is accompanied by a reversible stabilization of brain dynamics. That stabilization causes the critical oscillations, seen in the awake brain, to decrease during anesthetic-induced unconsciousness.
Fagerholm et al., 2016	3 mice receiving pentobarbital as an anesthetic	Optic voltage imaging	Neuronal avalanche	• Mice under anesthesia have decreased information capacity and decreased information transmission across brain regions. • While under anesthesia, neural activity also loses its scale-free attribute, indicating a deviation from criticality.
Hudetz et al., 2016	12 male rats receiving desflurane as an anesthetic	CAP measured by nLFP	Neuronal avalanche	• The presence of power–law distributions of CAPs with a slope of -1.5, an indicator of self-organized criticality, is found under deep anesthesia. • The distribution begins to deviate from power–law, thus deviating from criticality, with the recovery of consciousness.
Hahn et al., 2017	4 cats and 1 monkey receiving isoflurane as an anesthetic	32-channel electrode arrays/LFP	Neuronal avalanche	• Neuronal dynamics in the primary visual cortex follow a power-law distribution in the awake state as well as in anesthesia.
Krzemiński et al., 2017	4 monkeys receiving either ketamine-medetomidine, only ketamine, only medetomidine or propofol as an anesthetic	ECoG	DFA	• There is a decrease of LRTCs during loss of consciousness due to anesthesia.• • Brain regions characterized by strongest LRTCs during wakefulness exhibit the greatest decreases in LRTCs during anesthesia.
Stramaglia et al., 2017	20 human subjects receiving propofol as an anesthetic	fMRI	Structure-function relation	• The structure-function relation is strengthened under anesthesia compared to wakefulness. Authors put forward that the functional network is less dependent on the structural network at criticality, suggesting that anesthesia reflects a deviation from criticality.
Fagerholm et al., 2018	1 mouse receiving pentobarbital	Optic voltage imaging	Neuronal avalanche	• Using artificial neural networks, the authors showed that neuronal avalanches under anesthesia fit better a model connecting spatial and temporal information simultaneously. This model is less complex than in wakefulness, where spatial and temporal information are treated separately. The authors suggest that this reduction of complexity comes with a deviation from the critical state.
Fekete et al., 2018	3 rats anesthetized with urethane, 3 cats anesthetized with medetomidine and 2 monkeys anesthetized with ketamine, diazepam and remifentanil	Optic voltage imaging	Neuronal avalanche	• Wakefulness is associated with slightly subcritical behavior, whereas anesthetized rats can be supercritical or subcritical.
Kim and Lee, 2019	7 healthy human subjects receiving sevoflurane as an anesthetic	hdEEG	PCF	• PCF and Φ (measure of integrated information) are at their lowest during anesthetic-induced unresponsiveness. • Authors found a direct relationship between Φ, criticality and level of consciousness. They propose that anesthesia induces a significant deviation from criticality.
Lee et al., 2019	30 healthy human subjects anesthetized under isoflurane and 15 healthy human subjects anesthetized under ketamine	EEG	PLE	• Anesthetic-induced unconsciousness shows distinct PLE topography compared to that of conscious wakefulness and of DOC. • However, authors interpret this result by mentioning that this does not rule out the possibility that the brain is still functioning in a critical state in a DOC.
Varley et al., 2020b	1 monkey with 128-electrode arrays on the entirety of the left hemisphere receiving propofol or ketamine	ECoG	Neuronal avalanche	• Propofol dramatically restricts the size and duration of avalanches and presents a reduction in complexity, thus deviating from criticality. • Ketamine does not seem to induce significant changes in neuronal avalanches or brain complexity.
Kim M. et al., 2021	18 healthy human subjects receiving isoflurane anesthesia and 26 healthy human subjects receiving ketamine anesthesia	hdEEG	PCF	• Anesthesia causes a reduction in PCF, and thus a deviation from criticality.
Ponce-Alvarez et al., 2022	5 monkeys receiving either propofol, ketamine or sevoflurane as anesthetics	fMRI	Scaling parameter	• Study showed a phase transition from critical dynamics in the awake state to supercritical dynamics in the anesthetized state.
Pullon et al., 2022	16 healthy human subjects receiving propofol as an anesthetic	EEG	LRTCs	• Around loss of responsiveness, there is a transient increase in long time scale mutual information, followed by an abrupt loss of correlation, which indicates a deviation from criticality. • Around loss of responsiveness, a small increase in propofol concentrations causes a rapid collapse of long-time scale power envelope connectivity.

CAP, contiguous activity patterns; DFA, detrended fluctuation analysis; DOC, disorders of consciousness; ECoG, electrocorticography; EEG, electroencephalography; fMRI, functional magnetic resonance imaging; hdEEG, high-density electroencephalography; HP, hippocampus; LFP, local field potential; LRTCs, long-range temporal correlations; MEA, multielectrode arrays; nLFP, negative local field potential; PCF, pair correlation function; PLE, phase-lag entropy; S1, primary somatosensory cortex; V1, primary visual area.

Several studies have also investigated criticality in anesthetized animals, and contradicting results seem to emerge. Three studies investigated neuronal avalanches in mice under pentobarbital, and all showed a deviation from the critical state, either through an absence of power-law dynamics in neuronal avalanches (Scott et al., 2014; Fagerholm et al., 2016), or a reduction of complexity (Fagerholm et al., 2018). In studies investigating criticality in cats, monkeys and rats under various anesthetics (cats: medetomidine and ketamine; rats: urethane; monkeys: ketamine, diazepam and remifentanil), neuronal avalanches showed a deviation from criticality by losing their power-law properties under anesthesia (Fekete et al., 2018). However, another study looking at the effects of isoflurane on cats showed dynamical changes in the visual cortex suggesting that anesthesia may induce a state closer to criticality than what is measured in wakefulness (Hahn et al., 2017). Contradicting results can also be found in studies on rats. While under the effects of anesthesia using ketamine-xylazine and analyzing neuronal avalanches, it was found that the avalanches were losing their power-law properties (Ribeiro et al., 2010), while another study using desflurane showed the contrary: a preservation of power-law properties followed by a deviation from criticality during the recovery of consciousness (Hudetz et al., 2016). While measuring criticality using detrended fluctuation analysis and stability of neuronal dynamics in monkeys under propofol, ketamine or medetomidine, other studies have showed a decrease in long-range temporal correlations (Krzemiński et al., 2017) and a stabilization of neuronal dynamics (Solovey et al., 2015), both indicators of a deviation from criticality. Conversely, a more recent study on monkeys showed that propofol drastically reduced the size and duration of neuronal avalanches, while ketamine induced more wake-like dynamics (Varley et al., 2020b). Finally, a study using a statistical models incorporating a scaling parameter on fMRI brain measures of monkeys under either propofol, ketamine or sevoflurane showed that brain dynamics under anesthesia were supercritical when compared to resting brain activity (Ponce-Alvarez et al., 2022). The authors conclude that anesthetics have a disconnecting effect on particular brain regions (i.e., insular, cingulate, and parietal cortices), making them less potent at transmitting information to the system as a whole. Overall, the current literature in animal models of anesthesia shows contradictory results, and the anesthetics used in these studies seems to have varying effects on brain criticality.

### Coma and DOC

Coma and DOC are typically caused by a severe brain injury or brain dysfunction. Coma is a deep state of unconsciousness that lacks both wakefulness and awareness, and in which responses to stimulation and pain are completely absent (Blume et al., 2015). In DOC, however, awareness is lacking despite the preserved capacity for wakefulness, embodying a disconnect between wakefulness and awareness. In unresponsive wakefulness syndrome (also known as vegetative state), patients are able to awaken (eye opening), but show no behavioral signs of being aware of themselves or their surroundings, therefore lacking goal-oriented or willful behaviors (Multi-Society Task Force on PVS, 1994). As such, patients in unresponsive wakefulness syndrome are considered to be unconscious. Minimally conscious state presents with eye opening (wakefulness) and some reproducible, though minimal, oriented and/or willful behaviors (e.g., visual tracking, inconsistent command following) (Giacino et al., 2002). Coma and DOC can be temporary, but the neural and functional mechanisms by which their reversal is possible are unknown. In this population, assessing conscious awareness and establishing a prognosis for recovery in the absence of behavioral responsiveness are fundamental shortcomings of clinical practice. Ultimately, improving DOC patient diagnosis and prognosis are necessary to meaningfully contribute to clinical management and decision-making, leading to better-adapted rehabilitative strategies, and improved outcomes.

Even though criticality could be an interesting way to refine the diagnosis and prognosis of DOC, a very restricted number of studies have investigated this subject (Table 5). Most studies regrouped DOC as a single category, all of which showed a deviation from criticality using neuronal avalanches (Fekete et al., 2021), pair correlation function (Kim M. et al., 2021), phase lag entropy (Lee et al., 2019) or dimensionality (rate of decay in information transfer in regard to distance) (Abeyasinghe et al., 2020). A single study focused specifically on unresponsive wakefulness syndrome, and described the power-law distributions of network hubs. This study showed that the network loses its power-law properties in unresponsive wakefulness syndrome, indicating a deviation from criticality. This also represents a potential marker to be included in clinical practices relating to diagnosis of unresponsive wakefulness syndrome (Liu et al., 2014).

**TABLE 5:** Results of studies on criticality in coma and DOC.

References	Population	Cerebral activity measure	Criticality measure	Main findings
Liu et al., 2014	7 human participants with UWS as well as 7 healthy participants undergoing propofol sedation	fMRI	Scale-free distributions of functional node size and degree	• Study shows an absence of scale-free distribution of node degree in UWS patients, whereas it was still present during propofol sedation in healthy individuals. This indicates the absence of self-organizing processes necessary for criticality in UWS.
Lee et al., 2019	42 human participants with DOC (27 UWS, 15 MCS)	EEG	PLE	• The DOC brain shows distinct PLE topography compared to that of the conscious brain and of the anesthetized brain. • However, authors interpret this result by mentioning that this does not rule out the possibility that the brain is still functioning in a critical state in a DOC.
Abeyasinghe et al., 2020	13 human participants with DOC (6 UWS, 7 MCS), compared to 25 healthy human controls	PET/MRI	Dimensionality	• Dimensionality is higher in DOC patients than in controls, indicating that information decays faster in the DOC brain than in the healthy conscious brain. • Because a faster rate of decay in DOC patients across the same distance is observed, authors suggest that this difference arises from pathology in something other than the brain’s structural connections.
Fekete et al., 2021	171 human participants with DOC (77 UWS, 70 MCS, 24 EMCS), compared to 12 healthy human controls	EEG/MEG	Neuronal avalanche	• There is an attenuation of the slope of neuronal avalanches in DOC when compared to controls. The fact that the neuronal avalanches lose their power-law properties suggests a deviation from criticality.
Kim M. et al., 2021	25 human participants with DOC (9 UWS, 16 MCS)	hdEEG	PCF	• DOC patients have a lower PCF than healthy individuals, thus showing a deviation from criticality.

DOC, disorders of consciousness; EEG, electroencephalography; EMCS, emerging minimally conscious state; fMRI, functional magnetic resonance imaging; hdEEG, high-density electroencephalography; MCS, minimally conscious state; MEG, magnetoencephalography; PCF, pair correlation function; PLE, phase-lag entropy; UWS, unresponsive wakefulness syndrome.

### Seizures and epilepsy

Epilepsy is a brain condition characterized by a lasting predisposition to generate spontaneous seizures (Thijs et al., 2019). The development of epilepsy is said to result from an imbalance between excitatory and inhibitory activity in a particular neuronal network, disrupting neuronal processing and potentially affecting other neuronal networks (Fisher et al., 2005). Epilepsy can be defined as generalized when the epileptogenic networks are distributed across the whole brain and involve thalamocortical structures bilaterally, or can be defined as focal, where the epileptogenic network is usually located at the limbic or neocortical level in a single hemisphere (Fisher et al., 2005). A considerable part of research on epilepsy focuses on the localization of the epileptic focus of debilitating, pharmaco-resistant epilepsy to eventually proceed to a surgical intervention aiming at removing the epileptic focus (Rugg-Gunn et al., 2020). Considering that these gravely epileptic patients often have to go through an EEG evaluation prior to their operation (Chen and Koubeissi, 2019), several studies have investigated criticality in epilepsy (Table 6).

**TABLE 6:** Results of studies on criticality in epileptic seizures.

References	Population	Cerebral activity measure	Criticality analysis	Main findings
Cranstoun et al., 2002	7 human patients with temporal lobe epilepsy implanted with depth electrodes during evaluation for epilepsy surgery	iEEG	Neuronal avalanche	• Neuronal avalanches in patients with temporal lobe epilepsy follow power law distributions, providing support for self-organized criticality in epileptic humans.
Worrell et al., 2002	7 human patients with temporal lobe epilepsy who were implanted with depth electrodes during evaluation for epilepsy surgery	iEEG	Neuronal avalanche	• The probability of IEA and the time between successive IEA exhibit power-law scaling, showing evidence of self-organizing criticality in epileptic brains.
Cerf et al., 2004	6 epileptic human child participants with chronically implanted electrodes prior to surgery	SEEG/EEG	Parameter of fluctuation of electropathophysiological activity	• A high order parameter characterizing high electropathophysiological activity is found prior to seizures.
Parish et al., 2004	5 human patients with exclusively unilateral mesial temporal lobe seizures with chronically implanted electrodes prior to surgery	Continuous iEEG	DFA	• The epileptogenic (seizure-generating) hippocampus shows greater LRTCs compared to the non-epileptogenic contralateral hippocampus. • Study did not find any evidence for a change in LRTCs as seizures approached.
Li et al., 2005	12 rats with iEEG in temporal and frontal lobe on which epileptic seizures were induced with bicuculline	iEEG	Neuronal avalanche/LRTCs	• The pre-ictal state is accompanied by an increase in LRTCs. • An epileptic seizure could be considered as a ‘critical phenomenon’, culminating in a large event that is analogous to a ‘critical point’.
Monto et al., 2007	5 human patients with refractory neocortical epilepsy and intracranial electrodes implanted as part of the presurgical evaluation	iEEG	DFA	• Through interictal recordings, abnormally strong LRTCs near the seizure area are found. • Lorazepam attenuates beta-band LRTCs near the epileptic focus, whereas it strengthens LRTCs in other cortical areas.
Meisel et al., 2012	8 human patients undergoing surgical treatment for intractable epilepsy	ECoG	PLI	• During epileptic seizure, neuronal activity patterns deviate from the power-law distribution characterizing critical dynamics.
Arviv et al., 2016	20 human participants with refractory epilepsy being treated with AED compared to 18 healthy controls	MEG	Neuronal avalanche	• Epilepsy patients tend to exhibit larger neuronal avalanches, particularly during IEA. • Authors suggest that epilepsy is associated to substantially higher values than the expected theoretical values for a critical process.
Yan et al., 2016	3 human participants with refractory temporal lobe epilepsy	ECoG	Hurst exponent	• Intraictal activity is characterized by an increase in the Hurst exponent, suggesting higher stability and a supercritical state. • In preictal activity, the Hurst exponent changes in most channels across different brain areas, rather than restricted to the epileptic foci. Authors suggest that epileptic seizures may not result from activity at a single site, but rather a breakdown of the whole neuronal network activities.
Meisel, 2020	17 epileptic human patients undergoing presurgical ECoG monitoring and receiving a treatment of AED	ECoG	Neuronal Avalanche/ACF	• Pharmacological treatment of epilepsy using AED results in a reduction of the slope of neuronal avalanches as well as ACF, indicating a shift toward the subcritical state.
Fekete et al., 2021	31 epileptic human patients	EEG	Neuronal Avalanche	• In states of compromised information processing such as the preictal state in epileptic patients, neuronal avalanche show a deviation from criticality.
Hagemann et al., 2021	20 human patients with medically intractable focal epilepsy implanted with depth electrodes in various regions (hippocampus, amygdala, parahippocampal cortex, entorhinal cortex)	Single-unit recordings	Branching parameter *m*	• Even in preictal and interictal recordings of epileptic subjects, the human cortex operates in a stable, slightly subcritical regime.

ACF, autocorrelation function; AED, antiepileptic drugs; DFA, detrended fluctuation analysis; ECoG, electrocorticography; EEG, electroencephalography; IEA, interictal epileptiform activity; LRTCs, long-range temporal correlations; MEG, magnetoencephalography; PLI, phase-lag index; SEEG, stereoelectroencephalography.

Results are presented in 3 categories: intraictal (during seizure), interictal (between seizures), and preictal (before seizure) recordings. Firstly, studies investigating intraictal criticality properties unanimously suggest that the brain is in a supercritical state during a seizure. Greater long-range temporal correlations, measured by the Hurst exponent, were seen in intraictal brain activity, suggesting a supercritical state (Yan et al., 2016). While looking at the power-law of phase-locking intervals (PLI), a measure of synchronicity in oscillators, it was shown that the distributions were deviating from their power-law properties during a seizure in the direction of a supercritical state (Meisel et al., 2012). A study on rats has shown similar results, pointing out that seizures could be interpreted as “a generalized kind of phase transition analogous to a kind of critical point” (Li et al., 2005) considering the presence of strong long-range temporal correlations and an increase in neuronal avalanches.

During interictal recordings, two studies showed that the brains of epileptic patients operate in a critical state, considering that neuronal avalanches follow a power-law (Cranstoun et al., 2002; Worrell et al., 2002). Another study that investigated the branching parameter obtained from intracranial electrodes showed the absence of significant differences in criticality between the epileptic focus and its contralateral counterpart in the brain during interictal recordings (Hagemann et al., 2021). Conversely, while comparing the LRTCs located in the epileptic focus to the long-range temporal correlations present in the symmetrically contralateral region of the brain, two studies showed that long-range temporal correlations, measured by detrended fluctuation analysis, were stronger in the epileptic focus during interictal recordings, thus indicating a supercritical state in that region (Parish et al., 2004; Monto et al., 2007). An important concept in epilepsy that was also studied under the scope of criticality is interictal epileptiform activity (IEA), an abnormal brain activity without apparent seizure (Arviv et al., 2016). Using MEG, it was found that neuronal avalanches were greatly increased during these periods of IEA, suggesting that the brain, and especially the potential epileptic focus, was in a supercritical state (Arviv et al., 2016).

In terms of preictal activity, one study showed that preictal activity shows slightly subcritical traits, similar to that of wakefulness in the healthy brain (Hagemann et al., 2021). Another study that used a parameter of fluctuation of electropathophysiological activity to measure criticality suggests that preictal brain activity was in a critical state (Cerf et al., 2004). However, authors did not compare preictal activity to interictal activity for all patients, making it difficult to establish whether the critical state they claim to observe is distinct from the interictal brain or the healthy brain. Another study showed that preictal neuronal avalanches deviate from a power-law distribution, suggesting a deviation from criticality without specifying the direction (Fekete et al., 2021).

### Psychedelics and shamanic state of consciousness

Psychedelics are psychoactive drugs known for their capacity to trigger deeply profound existential experiences (Carhart-Harris, 2019). Though psychedelics seem to have had their greatest impact on Western culture starting in the 1960s, potential new medical applications of psychedelics in the fields of psychiatry and neurology have generated a revival of interest for these substances in the last decade. As agonists of 5-HT2A serotoninergic receptors, psychedelics are said to induce greater plasticity at the cortical level, where 5-HT2A receptors are most densely concentrated (Beliveau et al., 2017; Kraehenmann et al., 2017). The fact that they operate at the cortical level, said to be the basis of higher-level operations, is coherent with the fundamental changes in consciousness reported by psychedelic users. Interestingly, the shamanic state of consciousness is said to induce behavioral and conscious aspects similar to those seen under the influence of psychedelics, such as mystical experiences and feelings of disembodiment (Krippner, 2000; Winkelman, 2013; Sweeney et al., 2022).

Though only a few studies have investigated the effects of psychedelics on criticality using varying methodological approaches, the current state of the literature seems to suggest that psychedelics improve criticality in the brain (Table 7). One study looking at the ensemble of individual harmonic brain states under LSD found that the power-law properties of some of the frequency specific harmonics were closer to criticality than in the slightly subcritical resting brain (Atasoy et al., 2017). Another study, looking this time at both LSD and psilocybin, used the fractal dimension of cortical brain activity in spatial and temporal domains to establish criticality, considering that supporting evidence suggests that as a system approaches the critical point, it starts to organize itself as a fractal system (Watanabe et al., 2015; Varley et al., 2020a). This research group showed that both psychedelics drugs increased the fractal dimension of functional connectivity networks, therefore bringing the brain closer to criticality (Varley et al., 2020a). A study looking at the effects of ketamine as a psychedelic (a dose insufficient for ketamine to be considered an anesthetic) found a significant reduction in the pair correlation function, suggesting a deviation from criticality (Kim M. et al., 2021). A single study was found to describe the shamanic state and its impact on brain criticality. Concerning brain criticality during the shamanic state, results were strikingly similar to those seen during psychedelic usage; that is, pair correlation function was higher in beta and gamma bands when compared to controls, indicating a state closer to criticality than normal wakefulness (Huels et al., 2021).

**TABLE 7:** Results of studies on criticality in psychedelic and shamanic states consciousness.

References	Population	Cerebral activity measure	Criticality analysis	Main findings
Atasoy et al., 2017	12 healthy human participants receiving either LSD or a placebo either while listening to music or after listening to music	fMRI	Power-laws of brain harmonics	• LSD usage is associated with a closer fit of brain harmonics to power-laws as well as a slight change in the critical exponent, indicating a shift of brain dynamics toward criticality. • Even though brain dynamics in LSD and placebo both reside close to criticality, the induction of LSD tunes brain dynamics further toward criticality.
Varley et al., 2020a	LSD: 20 healthy participants undergoing 2 brain scans and receiving a placebo on one of the scans and LSD on the other one Psilocybin: 15 healthy participants undergoing brain scan while receiving psilocybin	fMRI	Fractal dimension of cortical brain activity in spatial and temporal domains	• Both psychedelic drugs (LSD and psolicybin) significantly increased the fractal dimension of functional connectivity networks. • LSD significantly increased the fractal dimension of BOLD signals, while psilocybin showed a non-significant trend in the same direction. • These results suggest that psychedelics are associated with evolution toward a critical state.
Huels et al., 2021	24 experienced shamanic healing practitioners compared to 24 control	EEG	PCF	• Shamanic trance increases PCF in beta and gamma bands, implying that this state of consciousness shifts the brain dynamics closer to criticality.
Kim M. et al., 2021	15 healthy participants receiving a psychoactive dose of ketamine considered too low to induce anesthesia	hdEEG	PCF	• PCF is significantly reduced in a psychedelic experience induced by ketamine, thus showing a deviation from criticality.

BOLD, blood-oxygen level dependent; EEG, electroencephalography; fMRI, functional magnetic resonance imaging; hdEEG, high-density electroencephalography; LSD, lysergic acid diethylamide; PCF, pair correlation function.

### Delirium

Delirium is an acute state of reduced awareness with disturbances in attention and cognition, which usually emerges over a short period of time in vulnerable patients (Hshieh et al., 2018). Even though the pathophysiological mechanisms of delirium are still poorly understood, several biological and psychological factors such as dementia, history of alcohol abuse, psychoactive medication use and advanced age (> 70 years) are often involved in the development of this medical condition (Hshieh et al., 2018). Delirium can present various behavioral characteristics, such as in the hypoactive form where the patient is lethargic, in the hyperactive form where the patient is agitated and can sometimes present hallucinations, and a mixed form where the patient fluctuates between agitation and lethargy. The fact that delirium presents such a broad range of behaviors makes it a difficult condition to diagnose making the study of criticality in this ASC particularly interesting as it could be used as a neurophysiological diagnostic biomarker. Furthermore, given that delirium is a serious medical complication with rapid onset, objective measures are necessary to better predict and prevent its onset.

Only a single criticality study was found relating to delirium (Table 8), which investigated long-range temporal correlations with autocorrelation function in delirious patients in post-operative care (Kim H. et al., 2021). Authors suggest a deviation from criticality in delirium, due to reduced autocorrelation function in the alpha band in delirious patients compared to non-delirious patients. Interestingly, this study also showed that caffeine helped to prevent delirium by bringing the cortical dynamics closer to the critical state (Kim H. et al., 2021).

**TABLE 8:** Results of studies on criticality in delirium.

References	Population	Cerebral activity measure	Criticality analysis	Main findings
Kim H. et al., 2021	11 delirious participants, 4 receiving caffeine for pain after surgery and 7 receiving a placebo	EEG	ACF	• ACF in the alpha band is significantly reduced in delirious participants, meaning that postoperative delirium may cause deviations from criticality. • Participants randomized to the caffeine condition demonstrate increased alpha ACF, thus increased criticality, concurrent with reduced delirium incidence.

ACF, autocorrelation function; EEG, electroencephalography.

### Meditation

Meditation is a mindfulness practice, originally seen in the Buddhist and Hindu cultures and introduced more recently in the Western culture, where the individual intentionally increases their awareness to thoughts and feelings (Basso et al., 2019). Several forms of meditation exist, two of the most popular being the open monitoring meditation, where the practitioner tries to monitor in a non-judgmental way everything that spontaneously comes into conscious awareness (Davidson and Lutz, 2008; Basso et al., 2019), as well as the focused attention meditation, where the individual tries to concentrate its attention on a single object or movement (ex: its own breath) while ignoring internal and external distractions (Yoshida et al., 2020). As described earlier, consciousness is said to be composed of arousal as well as awareness, making meditation (a practice where awareness is increased), a sort of “super-conscious” state.

Research on brain criticality in meditation is sparse (Table 9), and all studies found in this review investigated criticality in focused attention meditation. One study used detrended fluctuation analysis to measure long-range temporal correlations in experienced practitioners of meditation and found a reduction in long-range temporal correlations during meditation when compared to an eyes-closed resting control condition (Irrmischer et al., 2018). Still, it is difficult to establish that this deviation from criticality is purely due to the fact that the person is in a meditative state, as various studies have stated that experienced practitioners of meditation have permanent functional changes in their brain (Cahn et al., 2010; Dürschmid et al., 2020). To answer this blind spot in the literature, another study isolated the effects of the meditative state by looking at the brain criticality of novice practitioners of focused attention meditation who were instructed and guided through a meditation by an expert in the field. While using MEG data filtered in high frequency bands (> 100Hz) to look at neuronal avalanches, they stated that the neuronal avalanches of novices in a meditative state followed a power-law distribution with an exponent closer to the critical state compared to mind-wandering controls and compared to a rest state (Dürschmid et al., 2020).

**TABLE 9:** Results of studies on criticality in meditation.

References	Population	Cerebral activity measure	Criticality analysis	Main findings
Irrmischer et al., 2018	8 healthy experienced meditation practitioners compared to 11 healthy meditative-naïve controls	hdEEG	DFA	• In meditation practitioners but not in controls, focused attention meditation strongly suppresses LRTCs of neuronal oscillations relative to eyes-closed resting state. • Sustained meditation practice affects normal waking brain dynamics beyond the meditative period, such that experience meditation practitioners show increased LRTC during eyes-closed resting state compared to meditative-naïve controls.
Dürschmid et al., 2020	17 meditation-naïve healthy subjects participating in focused attention meditation	MEG	Neuronal avalanche	• Mindfulness meditation shifts scale-free dynamics toward the critical point by reducing neural noise. • The authors suggest that self-regulated attention in the form of mindfulness meditation may serve as a control parameter of criticality in the scale-free brain dynamics.

DFA, detrended fluctuation analysis; hdEEG, high-density electroencephalography; LRTCs, long-range temporal correlations; MEG, magnetoencephalography.

## Discussion

**FIGURE 3:**
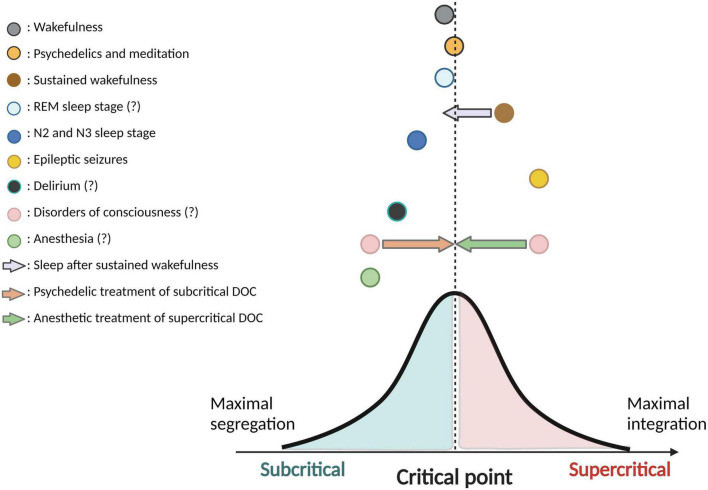
Directions of deviation from criticality in various ASC. This figure is a synthesis and schematic representation of the current findings from the literature on criticality in altered states of consciousness (ASC). Colored circles illustrate the various ASC and their hypothetic position relating to the critical state (or “critical point”). ASC located on the left side of the critical point have been shown (in minimally one study) to reflect a subcritical state, whereas ASC on the right side of the critical point have been shown (in minimally one study) to reflect a supercritical state. Distance from the critical point is schematic and not reflective of specific comparisons across ASC. Colored arrows represent a potential neuromodulation of criticality that a specific ASC could induce on another ASC. The bell-shaped curve is used to demonstrate a perfect balance between integration and segregation at the critical point. In the legend, question marks following the name of a particular ASC reflect that contradicting results have been found in the literature with regards to the subcritical/supercritical nature this ASC, and/or that findings remain sparse in this regard. Created with BioRender.com.

This scoping review aimed to map the current state of the literature on criticality across ASC, in order to appraise how the criticality hypothesis of consciousness is currently supported in the literature. Firstly, we found that the majority of studies published to date focus on criticality in anesthesia, sleep and epilepsy. Importantly, our scoping review of the literature confirmed that scientific evidence remains relatively scarce and methodologically inhomogeneous. Despite this major limitation, we also found evidence suggesting that most ASC presented show a deviation from the critical state, strengthening the hypothesis that deviations from criticality accompany ASC. Even if the direction of this deviation was rarely characterized in the articles presented in this review, growing evidence supports that NREM sleep reflects a subcritical state, epilepsy reflects a supercritical state, and psychedelics bring cortical dynamics closer to the critical state than what is observed in the healthy awake brain, which is slightly subcritical (refer to Figure 3 for the visual presentation of the results of this review). We also found that neuronal avalanches and long-range temporal correlations were the most commonly used methods to measure brain criticality in human and animal studies.

Although the study of brain criticality in ASC has seen a surge of publications in recent years, some limitations still persist in the current literature. First, knowing that brain criticality is a somewhat new concept in neuroscience, there is not yet a single definition or conceptual framework used to study criticality, making comparison between articles difficult. Second, a plethora of methods exist to characterize criticality in the brain, also rendering difficult the comparison of various studies. Indeed, two articles investigating criticality in the same ASC but using a different measure could come to divergent results, simply due to varying methodological approaches. In order to create a reliable criticality-based conceptual framework of ASC, future studies should assess criticality across a wide range of ASC using consistent methodology that can capture the amplitude and direction of deviations from the critical state. Third, the terminology related to the field of criticality is both complex and vast, making it difficult to scope the entirety of the work related to the concept in the literature. Some studies have investigated criticality-related measures or concepts without ever using the term “criticality”, rendering their papers less visible to the field, and undetected by the search strategy employed in the present review. Efforts should be made in the field of criticality to use more uniform terminology, in order to make it easier to find studies of interest and to ensure more efficient discourse across studies. Fourth, the intricate terminology related to criticality comes with downsides, such as the conflation of different concepts into a single term (e.g., avalanches used for neuronal avalanches as observed in local field potentials, but also used in the context of power-law of oscillations, as seen in more “whole-brain” approaches such as EEG and fMRI). Mixing up data coming from whole-brain methodologies, such as EEG and fMRI, to more spatially localized methods such as local field potentials can also be somewhat problematic. Indeed, when using whole-brain approaches, criticality becomes a one-dimensional measure applied to an extremely complex system. Evidently, this comes with a loss of information at both the spatial and temporal levels. It would be interesting to study criticality as a fluctuating metric across these dimensions as it would be a more accurate depiction of the brain’s complex dynamics. Finally, the application of criticality to the brain remains somewhat debated. Some have called into question the current methods used to assess criticality in the brain, claiming they are insufficient to establish or distinguish criticality in a multitude of systems, and pointing out the ubiquity of critical properties in varying neural systems with radically distinct properties (Beggs and Timme, 2012; Beggs, 2015, 2022; Destexhe and Touboul, 2021).

### Criticality as a framework for the diagnosis and neuromodulation of ASC

The healthy awake brain seems to operate very near the critical state (Pearlmutter and Houghton, 2009; Beggs and Timme, 2012; Beggs, 2015, 2022; Massobrio et al., 2015; Shi et al., 2022) rendering it most flexible and adaptable to its changing environment and processing demands. More specifically, it is thought to operate in a slightly sub-critical state, closer to chaoticity than synchrony (Priesemann et al., 2013, 2014; Fekete et al., 2018; Toker et al., 2022). Proximity to criticality would enable the brain to benefit from most of the functional characteristics of criticality (e.g., optimal information processing, ability to reorganize efficiently) whilst protecting itself from tipping off toward the supercritical side, associated with large-scale synchronized activity often seen in epileptic seizures (Shew and Plenz, 2013; Priesemann et al., 2014; Beggs, 2022). As seen in this review, the ability of psychedelics to increase proximity to criticality (Beggs and Timme, 2012; Pullon et al., 2022), also suggests that healthy wakefulness may in fact not be exactly at criticality (Atasoy et al., 2017; Varley et al., 2020b).

Considering that the study of brain criticality under anesthesia has sprouted what seems to be the greatest amount of evidence across all ASC, it is reasonable to think that this has been done with various applications in mind. Indeed, in a scoping review on clinical applications of criticality, Zimmern points out two distinct clinical applications of criticality in anesthesia. The first being a marker of depth of anesthesia and the second being a predictive marker of recovery from a comatose state (Zimmern, 2020). Given that having a reliable marker of unconsciousness under anesthesia could help reduce the prevalence of intraoperative awareness, a traumatizing event where a patient undergoing surgery is still aware of what is happening and can recall the events of the surgery (Mashour et al., 2012), various studies have begun emerging relating criticality to prediction of anesthetic depth (Alonso et al., 2014; Krzemiński et al., 2017; Thiery et al., 2018). The prediction of recovery in DOC or comatose patients using criticality is also an emerging area of research (Zimmern, 2020). Future studies should look into more ways to clearly distinguish criticality-related traits between anesthesia and DOC, to 1) help with the diagnosis and prognostication of DOC and 2) explore how sedation and/or anesthesia could potentially improve criticality in coma and DOC patients. Indeed, though highly counter-intuitive, anesthesia (generally conceptualized as inducing a subcritical state) could potentially be used to improve criticality in DOC patients whose brains are in a supercritical state. Given that recent evidence suggests that DOC patients can be either in subcritical or supercritical state (Toker et al., 2022), treatment would have to be personalized according to each patient’s deviation from the critical state. As such, substances that are hypothesized to induce subcritical states, such as sedatives/anesthetics, could be used to treat supercritical states, while substances that improve criticality (e.g., psychedelics) could be used to treat pathological subcritical states. Evidently, additional studies are needed to further characterize the direction of deviations from criticality in various ASC before exploring such hypotheses.

Evidence in epileptic seizures (intraictal) seems to suggest a supercritical state, though results pertaining to interictal and preictal activity in epileptic patients are still unclear. Additional evidence supporting the fact that epileptic seizures reflect a supercritical state can also be deducted from the clinical applications and short-term treatments of epileptic seizures. Indeed, antiepileptic medications tend to induce a decrease in neuronal avalanches as well as in long-range temporal correlations, which suggests that their ability to decrease seizure occurrence may come from their ability to shift the brain toward a subcritical state (Meisel, 2020), as a way to counteract the supercritical functional properties of seizures. Treatment of refractory status epilepticus with strong doses of propofol may also reflect a similar phenomenon (Rai and Drislane, 2018). Though various biomarkers for epilepsy have been studied to this day, most of them showed contradicting and inconsistent results and almost none of them used brain criticality as a potential marker (Pitkänen et al., 2019). Further studies should investigate in greater depth how brain criticality fluctuates in epileptic patients, across pre-, intra-, and interictal activity, and in a baseline, resting state, compared to healthy controls.

While sleep has been more widely studied under the scope of criticality, the preliminary consensus that seems to emerge from the current literature is that NREM sleep presents a deviation from criticality, with preliminary results suggesting a deviation toward the subcritical side (Tagliazucchi et al., 2013). The fact that REM sleeps seems to preserve brain criticality is coherent with the fact that EEG activity during REM sleep resembles that seen in wakefulness (Simor et al., 2020), and that this sleep stage supports conscious-like experiences in sleepers, including non-lucid and lucid dreams (Jing et al., 2016). As REM sleep can be referred to as the most “conscious” stage of sleep, N3 could be referred to as the most “unconscious” stage, with high prevalence of slow-wave activity and minimal behavioral responsiveness. Though this has yet to be unanimously confirmed, N3 could therefore be expected to show the greatest deviation from criticality. Elucidating the functional characteristics and critical behavior across sleep stages could shed light on the restorative properties of sleep and its association to learning, memory, and neuroplasticity (Walker and Stickgold, 2006). Additionally, it could contribute to further our understanding of the detrimental effects of sleep deprivation, or sleep disorders, on the brain.

Research around psychedelics and criticality is still in its early phases, but results seem unanimous in suggesting that psychedelics bring cortical dynamics closer to the critical point than in healthy wakefulness. This finding could have important implications for the treatment of pathological ASC in which the brain operates in subcritical states. As previously mentioned, a recent article published after our review of the literature seems to suggest that DOC patients could either be in a super- or subcritical state (Toker et al., 2022). This highlights the importance of evaluating the brains’ critical state prior to choosing a potential course of treatment. When the brain of a DOC patient is in a subcritical state, psychedelics could potentially be used as a pharmacological neuromodulation technique, bringing the cortical dynamics closer to the critical state. Indeed, psychedelics are said to increase conscious content in awake individuals who experience a “psychedelic state” (Scott and Carhart-Harris, 2019). Also, as mentioned by Scott and Carhart-Harris (2019), brain complexity (measured by Lempel-Ziv compressibility, a measure of criticality) is positively correlated with states of awareness (Scott and Carhart-Harris, 2019), and their current hypothesis is that psychedelic-induced increases in brain complexity could also increase conscious content of DOC patients, thus inducing a faster recovery. Seeing as the standard conceptualization of consciousness includes two distinct mechanisms (i.e., *wakefulness* or “arousal” and *awareness* or “conscious content”), it could be argued that substances that stimulate arousal, like methylphenidate and other dopaminergic agents, could also optimize consciousness recovery in DOC patients. Indeed, some preliminary results linking dopamine to DOC have started to emerge (Ciurleo et al., 2013; Edlow et al., 2020; Spindler et al., 2021), but we argue that psychedelics could be used either as a complement or as an alternative to these dopaminergic agents. Considering that arousal alone is not sufficient to induce a recovery from DOC, by potentially improving criticality and awareness, psychedelics could be the key to effectively treating DOC patients. Further characterization of the criticality properties of both psychedelics and DOC are necessary prior to such investigations, but future experiments comparing the effects of dopaminergic stimulants to psychedelics in DOC patients could help us identify whether drugs that increase criticality and conscious content, like psychedelics are hypothesized to do, are better at treating DOC than drugs that stimulate arousal (Scott and Carhart-Harris, 2019).

Meditation and shamanic states of consciousness present interesting results in the way that they also bring the brain network closer to the critical state. Benefits of meditation have been vastly studied in the current literature. Among others, benefits relating to stress reduction, anxiety, depression and pain improvement (Brandmeyer et al., 2019) seem to consistently arise when introducing meditation protocols. While some studies have proposed a vast number of structural and functional correlates of the mechanisms by which meditation influences health-related outcomes, such as reduced activity in the Default Mode Network (Garrison et al., 2015) or increased cortical thickness in prefrontal and insular regions (Hölzel et al., 2011), we argue that brain dynamics converging toward the critical state could be a potential explanatory factor of beneficial outcomes of meditation. Considering that the brain working in a critical state is optimized for information processing as well as flexibility of thought, future research should aim to characterize the direct contribution of brain criticality on health-related positive outcomes of meditation.

Though our review did not seek articles on the effects of caffeine on the brain functional network, caffeine was found to bring the cortical dynamics closer to criticality in a study on delirium (Irrmischer et al., 2018). This is an interesting result, as it could possibly explain the increases in cognitive capacities, such as concentration and memory enhancement (Cappelletti et al., 2015), reported while under this substance. Various clinical applications could also emerge from this finding, especially considering that caffeine is such an easily accessible drug and that is widely accepted to be safe for consumption in various populations. Some researchers are already looking at the applications of caffeine on anesthesia emergence and preliminary results are promising, showing faster emergence from anesthesia in subjects who were injected with intravenous caffeine (Hölzel et al., 2011; Cappelletti et al., 2015). These results have been reported in rats (Fox et al., 2020) as well as in humans (Fong et al., 2018) without any adverse effects. When looking at these findings within the scope of criticality, it is logical to think that a substance bringing cortical dynamics closer to criticality (caffeine) could be used to restore consciousness in an ASC generally conceptualized to induce a subcritical state (anesthesia). With this logic in mind, applications of caffeine as a treatment for subcritical DOC could also be plausible, though further research is necessary to characterize the criticality properties of caffeine across a range of ASC.

### Study limitations

This scoping review should be interpreted in light of certain limitations. First, since the term “criticality” is a somewhat new concept in neuroscience, some studies looking at measures related to criticality (e.g., long-range temporal correlations, neuronal avalanches, detrended fluctuation analysis, Hurst exponent, and others) do not explicitly use the term “criticality”, making it possible that these articles were not found during the literature search. In future reviews on the topic of criticality, the search strategy should be adapted to reflect the state of the literature and include these measurement methods as key search terms to make sure that all related articles are found.

Second, the fact that different measurement methods (e.g., EEG, fMRI) and different types of subjects (humans and animals) were compared to one another in this review implies prudence in the interpretation of the results. In the light of this limitation, we suggest that future projects explore brain criticality in various ASC while using the same measurement methods of criticality. This will lead to a stronger conceptualization of criticality, making it clearer which states are sub-critical, near criticality or super-critical.

Third, this review did not include recent theoretical advances on criticality that focus on predicting a critical point and modulating brain criticality. It is theoretically known that the underlying mechanisms of criticality, such as the type of phase transition [e.g., first order (abrupt) and second order (gradual)] or the bifurcation structure (e.g., Trans-critical (smooth transition), Hopf (onset of oscillation), Fold (abrupt transition) bifurcation, etc.) at a critical point shape the transition pattern and the system stability, which are directly associated with loss and recovery of criticality under external stimuli (Scheffer et al., 2009; Bury et al., 2020) as well as the signal characteristics at a critical point. However, because of the generality of the criticality measures currently considered in this review, which are observable regardless of the type of phase transition/bifurcation structure, criticality measures alone cannot differentiate criticality that originated from different types of phase transition/bifurcation. Therefore, it may lack a precise evaluation of the deviation from criticality and result in inconsistent and variable outcomes in application of criticality measure to ASC.

Finally, criticality has been mainly applied to ASC to characterize and differentiate the brain states quantitatively and qualitatively. However, it does not explain how the brain can recover from ASC and return to a normal state, near the critical point. In the future, the scope of reviews should be expanded to include principle and mechanism-based studies such as dynamical system theory and critical transition that describe the state transition behaviors during the loss and recovery of criticality in the brain.

## Conclusion

This scoping review showed that the study of criticality in ASC is still in its infancy, and that the current literature lacks homogeneity in its assessment tools as well as it analytical models and approaches. In the future, by directly mapping brain criticality using homogeneous measurement methods across a range of ASC, it would be easier to establish clear and widely accepted directions of deviation from the critical state. Further research is particularly needed in the fields of sleep, DOC, anesthesia and delirium. Establishing these clear directions of deviation would not only help in the potential development of new diagnostic and treatment tools for pathological ASC, but also in helping understand the functional and neural basis of consciousness and its alterations.

## Author contributions

L-PB completed the literature search, wrote a report of the results in French, and completed Table 1. GV screened the first set of article and gave his insight on the review. CG revised the literature search and adapted L-PB’s report into a complete English article. CG wrote the first draft of the discussion and created all tables (except Table 1) and figures. UL gave his insight and recommendations on the review and helped with writing the manuscript. CD screened the first and second set of article, as well as managed and validated the entirety of the first and second phase of the manuscript. All authors revised the manuscript.
